# Is a more aggressive COVID-19 case detection approach mitigating the burden on ICUs? Some reflections from Italy

**DOI:** 10.1186/s13054-020-02881-y

**Published:** 2020-04-28

**Authors:** Giulia Lorenzoni, Corrado Lanera, Danila Azzolina, Paola Berchialla, Dario Gregori, COVID19ita Working Group, Dario Gregori, Corrado Lanera, Paola Berchialla, Dolores Catelan, Danila Azzolina, Ilaria Prosepe, Annibale Biggeri, Cristina Canova, Elisa Gallo, Francesco Garzotto, Giulia Lorenzoni, Nicolas Destro

**Affiliations:** 1 Unit of Biostatistics, Epidemiology and Public Health, Department of Cardiac, Thoracic, Vascular Sciences, and Public Health, University of Padova, Via Loredan, 18, 35131 Padova, Italy; 2 Department of Translational Medicine, University of Oriental Piedmont, Novara, Italy; 3 Department of Clinical and Biological Sciences, University of Turin, Novara, Italy

Italy is the first European country in which the COVID-19 epidemic outbreak has spread, starting from two regions in Northern Italy, Veneto, and Lombardia. The outbreak poses a relevant burden on hospital resources, with a marked increase in the intensive care unit (ICU) occupancy rates [1].

It has been hypothesized that the proportion of severe infections that need intensive care could be affected by the testing strategy. At the beginning of the epidemic outbreak (21 February), an extensive testing strategy of both symptomatic and asymptomatic subjects has been adopted in Veneto and Lombardia. However, soon after the starting of the outbreak (27 February), the Italian Ministry of Health introduced restrictions in testing asymptomatic/mild symptomatic subjects. Such a recommendation has been a topic of debate among Italian scientists and policymakers since it has been suggested that also asymptomatic patients seem to transmit the infection [2]. For this reason, different strategies have been adopted at the regional level, thanks to the local autonomy of the regional health services. The Lombardia region adopted the recommendation [1], while the Veneto region did not apply the restrictions in testing asymptomatic/mild symptomatic patients [3], in line with the strategy adopted by the Republic of Korea.

To compare such two testing strategies, we assessed the relationship between the percentage of ICU admissions on the resident population and the percentage of asymptomatic/mild symptomatic subjects tested on the resident population, in Lombardia and Veneto.

Analyses are based on official data [4]. The asymptomatic/mild symptomatic subjects tested were obtained by subtracting the daily number of newly hospitalized patients from the total number of tests performed on the same day. The choice of asymptomatic patients instead of the overall number of patients was performed to avoid artifactual collinearity in the two dimensions being analyzed. Smoothing approximation using a loess regression method using polynomials of degree 2 with the alpha parameter set to 1.5 [5] has been fitted. The results are reported in Fig. 1. At the beginning of the observation (24 February), the percentage of asymptomatic/mild symptomatic subjects tested was 0.014% in Lombardia and 0.044% in Veneto, with 0.00019% and 0.00008% admissions in ICU in Lombardia and Veneto, respectively. At the 27th of March, the asymptomatic/mild symptomatic subjects tested were 0.83% in Lombardia and 1.66% in Veneto, with 0.01283% and 0.00689% of subjects admitted to the ICU in Lombardia and Veneto, respectively. Such data show a higher percentage of asymptomatic/mild symptomatic subjects tested since the beginning of the outbreak in Veneto, corresponding to a lower percentage of subjects admitted to the ICU.

**Fig. 1:**
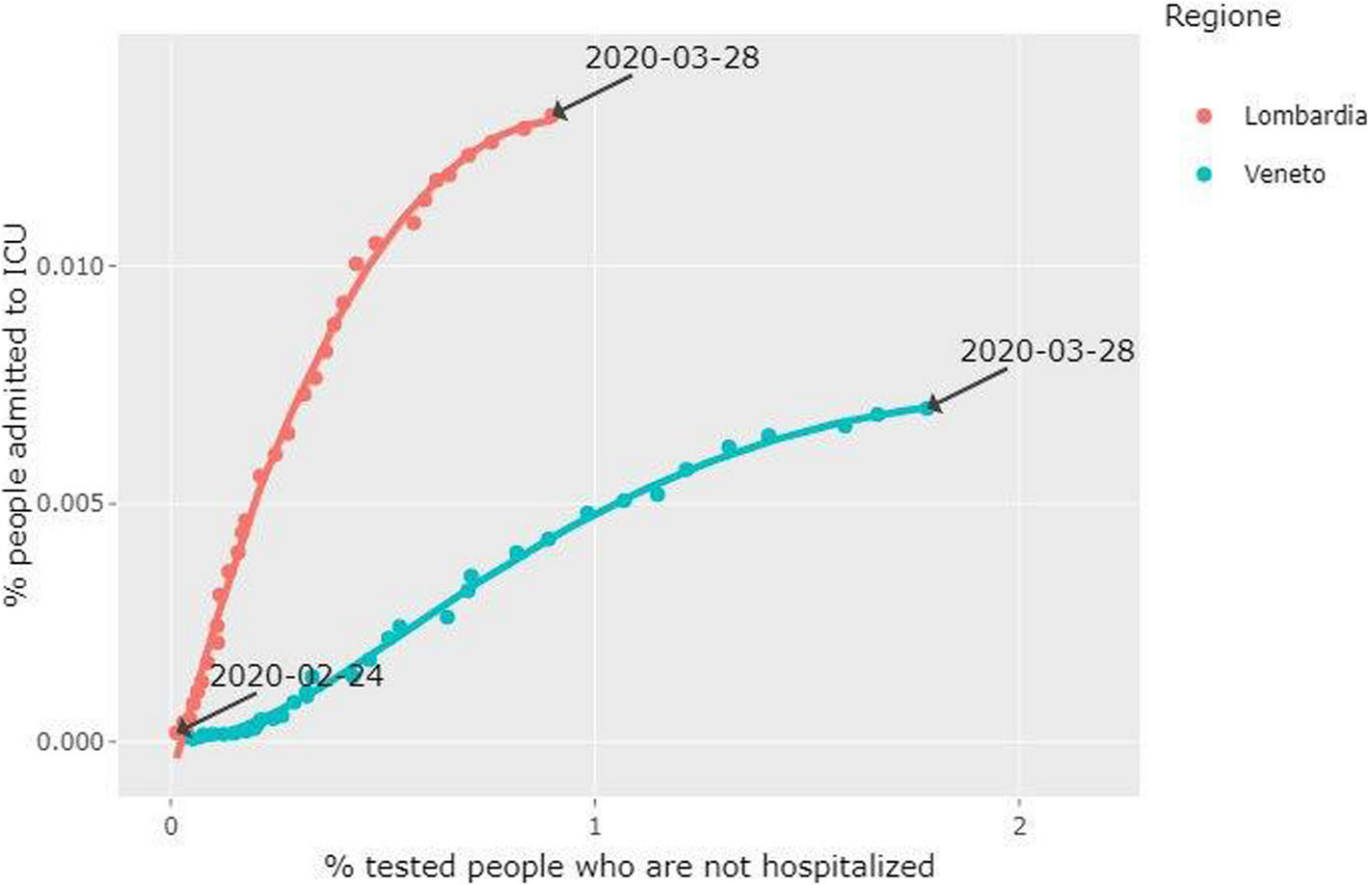
The *x*-axis reports the percentage (%) of subjects not hospitalized who underwent COVID-19 testing; the *x*-axis reports the percentage (%) of subjects admitted to the ICU (calculated on resident population). The continuous line indicates the local polynomial regression fitting; the dotted line shows the observed percentages

These findings suggest that testing also asymptomatic/mild symptomatic patients would help reduce the proportion of most severe cases eventually requiring ICU and thus limiting the risk of saturation of ICU units.

## Data Availability

The datasets used and/or analyzed during the current study are available from the corresponding author on reasonable request.

## References

[1] Grasselli G, Pesenti A, Cecconi M. Critical care utilization for the COVID-19 outbreak in Lombardy, Italy: early experience and forecast during an emergency response. JAMA. 2020. [Epub ahead of print]. 10.1001/jama.2020.4031 32167538

[2] Bai Y, Yao L, Wei T, Tian F, Jin D-Y, Chen L, et al. Presumed asymptomatic carrier transmission of COVID-19. JAMA. 2020. [Epub ahead of print]. 10.1001/jama.2020.2565 PMC7042844 32083643

[3] Pisano GP, Sadun R, Zanini M. Lessons from Italy’s response to coronavirus. Harvard Business Review. 2020 Mar 27; Available from: https://hbr.org/2020/03/lessons-from-italys-response-to-coronavirus. [cited 2020 Mar 30].

[4] {covid19ita}. covid19ita. Available from: https://r-ubesp.dctv.unipd.it/shiny/covid19ita/. [cited 2020 Mar 28].

[5] Harrell FE Jr. Regression modeling strategies with applications to linear models, logistic and ordinal regression and survival analysis (2nd Edition). Cham: Springer; 2015. http://biostat.mc.vanderbilt.edu/rms.

